# Exercise and Creatine Supplementation to Augment the Adaptation of Exercise Training Among Breast Cancer Survivors Completing Chemotherapy: Protocol for an Open-label Randomized Controlled Trial (the THRIVE Study)

**DOI:** 10.2196/26827

**Published:** 2022-04-01

**Authors:** Darpan I Patel, Angela Gonzalez, Crisann Moon, Monica Serra, Preston Blake Bridges, Daniel Hughes, Geoffrey Clarke, Lisa Kilpela, Rozmin Jiwani, Nicolas Musi

**Affiliations:** 1 Biobehavioral Research Laboratory School of Nursing University of Texas Health Science Center at San Antonio San Antonio, TX United States; 2 Barshop Institute for Longevity and Aging Studies University of Texas Health Science Center at San Antonio San Antonio, TX United States; 3 Mays Cancer Center University of Texas Health Science Center at San Antonio San Antonio, TX United States; 4 Institute for Health Promotion Research University of Texas Health Science Center at San Antonio San Antonio, TX United States; 5 Research Imaging Institute School of Medicine University of Texas Health Science Center at San Antonio San Antonio, TX United States; 6 Department of Psychiatry and Behavioral Sciences School of Medicine University of Texas Health Science Center at San Antonio San Antonio, TX United States

**Keywords:** rehabilitation, supplements, resistant exercise, oncology, quality of life, doxorubicin

## Abstract

**Background:**

In breast cancer survivors, chemotherapy-induced muscle loss has been shown to be attenuated with structured resistance exercise. Creatine supplementation can increase bioenergetics in skeletal muscle, which helps to improve overall strength and endurance and reduce muscular fatigue. Therefore, we hypothesize that adding creatinine supplementation to exercise training will accelerate improvements in strength, endurance, and bioenergetics in breast cancer survivors.

**Objective:**

The primary objective is to determine the effects of combining creatine supplementation with exercise on modulating strength and physical function in breast cancer survivors by comparing these effects to those of exercise alone. The secondary objectives are to determine if creatine supplementation and exercise can increase the intramuscular storage of creatine and improve body composition by comparing this intervention to exercise alone.

**Methods:**

We aim to test our hypothesis by conducting an open-label randomized controlled trial of 30 breast cancer survivors who have completed chemotherapy within 6 months of enrollment. Eligible participants will be equally randomized (1:1) to either a creatine and exercise group or an exercise-only group for this 12-week intervention. Individuals who are randomized to receive creatine will be initially dosed at 20 g per day for 7 days to boost the availability of creatine systemically. Thereafter, the dose will be reduced to 5 g per day for maintenance throughout the duration of the 12-week protocol. All participants will engage in 3 center-based exercise sessions, which will involve completing 3 sets of 8 to 12 repetitions on chest press, leg press, seated row, shoulder press, leg extension, and leg curl machines. The primary outcomes will include changes in strength, body composition, and physical function in breast cancer survivors. The secondary outcomes will be intramuscular concentrations of creatine and adenosine triphosphate in the vastus lateralis, midthigh cross-sectional area, and quality of life.

**Results:**

As of October 2021, a total of 9 patients have been enrolled into the study. No unexpected adverse events have been reported.

**Conclusions:**

Creatine is being studied as a potential agent for improving strength, endurance, and bioenergetics in breast cancer survivors following chemotherapy. The findings from our trial may have future implications for supporting breast cancer survivors in reversing the muscle loss experienced during chemotherapy and improving their physical function and quality of life.

**Trial Registration:**

ClinicalTrials.gov NCT04207359; https://clinicaltrials.gov/ct2/show/NCT04207359

**International Registered Report Identifier (IRRID):**

PRR1-10.2196/26827

## Introduction

### Background

Survivorship after a breast cancer diagnosis is multifocal; it includes long-term care planning that is coordinated with a survivor’s oncologist, primacy care provider, and social work support and should include an active lifestyle that is inclusive of exercise. Yet, individuals with breast cancer are at high risk for skeletal muscle wasting, which may be exacerbated by cancer treatment or tumor-related factors [1,2] and can negatively impact the ability to complete activities of daily living. Lower extremity muscle weakness is also associated with long-term fatigue in breast cancer survivors [3]. Studies on resistance exercise interventions have reported notable improvements in strength, endurance, and body composition in breast cancer survivors [4,5]; however, identifying strategies for enhancing adaptations to exercise among cancer survivors is of importance.

In the context of cancer survivorship, a large percentage of cancer survivors exhibit a loss of muscle mass that impacts their ability to perform activities of daily living [6]. The root causes of this loss of muscle mass are cancer treatment and tumor proteins [6,7]. Muscle wasting in patients with cancer, in combination with normal aging processes, dramatically increases the risk of disability and accelerates the aging process [8]. Certain cancer therapies can exacerbate this decline in physical function and result in disabilities that patients cannot independently manage [9]. Resistance training has been demonstrated to improve physical function, muscular strength, and endurance in breast cancer survivors. However, the relationship among body mass, treatment toxicity, and cancer recurrence requires those in the field of exercise oncology to identify modalities for promoting such exercise adaptations. Therefore, we hypothesize that adding creatine supplementation to a structured resistance training intervention can lead to better improvements than those resulting from exercise alone.

Creatine is a naturally occurring substance in the human body that is synthesized endogenously in the kidneys, pancreas, and liver from amino acids (ie, arginine, glycine, and methionine) at a rate of approximately 1 to 2 g per day [10]. An additional 1 g per day of creatine is typically consumed through the diet of those who eat meat and fish [11]. In total, the creatine pool in skeletal muscle averages at about 120 g for a 70-kg individual with a proposed creatine capacity of up to 160 g (via supplementation) [12].

Approximately 2 g of creatine are lost per day through urination. However, aging is associated with an incremental loss of intramuscular creatine stores [13,14]. Notably, creatine supplementation has been shown to reverse this loss [15]. Studies have demonstrated the efficacy of creatine supplementation in augmenting training adaptations and have reported positive outcomes, such as improved strength and physical function in a variety of healthy and clinical populations [11,12]. Although some publications suggest that creatine supplementation alone can increase strength and work capacity and delay the onset of fatigue in older men and women [16-19], the majority of studies suggest that the benefit of creatine supplementation is greatest when it is combined with hypertrophic stimuli, such as resistance exercise training [20].

Creatine supplementation has gained attention in the medical field because of the numerous health and quality of life benefits it has for people with muscular and neurological diseases, such as McArdle disease, Duchene dystrophy, myasthenia gravis, amyotrophic lateral sclerosis, and Parkinson disease, and its limited side effects [21]. Creatine is crucial to maintaining muscle energetics because of its role in rephosphorylating adenosine diphosphate to adenosine triphosphate (ATP). To date, few studies have examined the use of creatine supplementation to augment cancer treatment–associated declines in muscle mass and function [22-25]. Those that exist have generally failed to demonstrate its benefits for strength and function. A limitation to these studies is that most have investigated creatine supplementation alone. Only 1 study of cancer survivors has been published on combining creatine supplementation with resistance training, which resulted in improvements in lean body mass [22]. No such studies to date have been conducted on patients with breast cancer—a population that is particularly vulnerable to the loss of lean mass and function. Despite the benefits of exercise on reversing the anthracycline-related effects on muscle wasting, breast cancer survivors remain fatigued and deconditioned long into survivorship [26]. Therefore, there is a significant need to identify alternative strategies for reversing the known deleterious effects of chemotherapy in this population. The paucity of research in this area stems from the lack of awareness of the potential role that creatine supplementation plays in cancer survivors.

### Safety and Toxicity of Creatine

Creatine has not been evaluated by the Food and Drug Administration for safety; however, it is a widely used over-the-counter supplement. Creatine is one of the most highly studied supplements, and numerous studies have demonstrated its safety and tolerability [12,27]. In the four papers published to date on creatine supplementation in patients with cancer [22-25], only 2 of the 333 patients who were taking creatine reported any adverse effects (muscle cramping and mucus production) [22]. Other commonly identified side effects of creatine supplementation include nausea, diarrhea, dizziness, and gastrointestinal pain, but their severity is often mild [12,27].

### Study Objectives

The primary objective is to determine the effects of creatine supplementation, in combination with exercise, on modulating strength, body composition, and physical function in breast cancer survivors by comparing these effects to those of exercise alone. The primary hypothesis is that creatine supplementation will result in significantly greater gains in strength and physical function and improved body composition in breast cancer survivors compared to those resulting from exercise alone.

The secondary objectives are to determine if supplemental creatine can be used to increase intramuscular storages of creatine, alter energy storage, and improve quality of life. The hypothesis for these objectives is that creatine supplementation will significantly increase intramuscular concentrations of creatine and ATP in the vastus lateralis when compared to exercise-only controls. We also hypothesize that the creatine group will have significantly greater muscle cross-sectional areas and significantly lower levels of intramuscular fat compared to those of the exercise-only controls. Finally, we hypothesize that both groups will have a significantly better quality of life following the 12-week program.

### Trial Design

**Figure 1:**
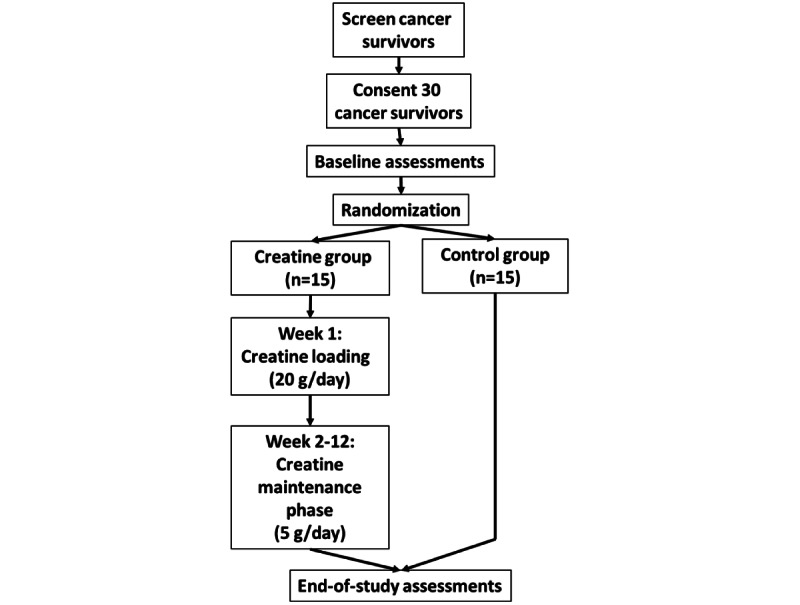
Individuals randomized to the creatine group will exercise 3 times per week for 12 weeks and take creatine in powder form as indicated. Individuals in the control group will exercise 3 times per week for 12 weeks without creatine dosing.

Our prospective study is a single-center, open-label randomized controlled trial. Figure 1 shows the study design, and Table 1 presents the schedule of events. This study protocol follows the SPIRIT (Standard Protocol Items: Recommendations for Interventional Trials) Statement recommendations [28].

**Table 1:** Study schedule of events.

Event	Screen day (−28 to −7 days)	Baseline assessments (−7 to −2 days)			Study week				12-week end-of-study assessments (>2 days and <7 days after the last exercise session)	
		Visit A	Visit B	Week 2		Week 5	Week 9	Visit A		Visit B
Phone screen	✓									
Physical exam	✓							✓		
Medical history	✓			✓		✓	✓	✓		
Medication history	✓			✓		✓	✓	✓		
Clinical labs	✓			✓				✓		
Electrocardiography	✓			✓						✓
Vital signs	✓							✓		
Height	✓									
Weight	✓							✓		
Body circumference	✓							✓		
Waist to hip ratio	✓							✓		
Mood disorder questionnaire										
Pregnancy test		✓						✓		
Handgrip dynamometry		✓						✓		
Isometric leg strength		✓						✓		
Dual x-ray absorptiometry		✓						✓		
6-minute walk test		✓						✓		
Survey completion		✓						✓		
Magnetic resonance imaging and magnetic resonance spectroscopy			✓							✓
1-repetition and 10-repetition maximum testing			✓							✓
Exercise familiarization			✓							
Dispense creatine (20 g/day)			✓							
Dispense Creatine (5 g/day)				✓		✓	✓			

## Methods

### Ethical Approval

This trial has been approved by the institutional review board at the University of Texas Health Science Center at San Antonio and the protocol review committee at the National Cancer Center Institute Mays Cancer Center at the University of Texas Health Science Center at San Antonio (2019-610H). All participants who meet the inclusion criteria will be eligible for participation. Any deviations from the protocol, breaches of confidentiality, and reportable adverse events will be reported to the institutional review board and Data Safety Monitoring Board at the Mays Cancer Center. The study is registered with ClinicalTrials.gov (trial number: NCT04207359).

### Study Setting

The study will enroll breast cancer survivors from San Antonio, Texas, and the surrounding community. The majority of the patients who will be enrolled in this study will be patients of the Mays Cancer Center at the University of Texas Health Science Center at San Antonio—a National Cancer Institute–designated cancer center. An interprofessional team of exercise physiologists, medical oncologists, nutritionists, mental health professionals, personal trainers, research coordinators, and research assistants will implement the various aspects of this study.

### Study Population

The study will enroll 30 patients with breast cancer who have completed chemotherapy within 6 months prior to consenting to this study. The recruitment methods include media advertisements, community events, and queries of institutional medical records for potentially eligible subjects. The inclusion criteria are the following: (1) an age of 18 to 75 years; (2) a diagnosis of breast cancer and the recent (within 6 months) completion of chemotherapy; (3) the willingness to attend 3 exercise sessions per week; (4) the ability to take oral medications; (5) the willingness and ability to provide consent for participating in the study; and (6) a serum creatinine level of ≤1.5 times the upper limit of normal or an estimated glomerular filtration rate of ≥30 mL/min/1.73 m^2^, as determined by the Chronic Kidney Disease Epidemiology Collaboration equation. The exclusion criteria are as follows: (1) physical indications that performing exercise may be limited or contraindicated; (2) poorly controlled hypertension (resting systolic blood pressure: >160 mm Hg; resting diastolic blood pressure: >95 mm Hg); (3) current tobacco use (within 6 months); (4) anabolic steroid use; (5) a pitting edema grade of 2+; (6) patients who are currently undergoing medical treatment for cancer (ie, currently undergoing chemotherapy; radiation therapy is allowed); (7) a history of moderate-severe heart disease (New York Heart Classification of >grade II) or pulmonary disease (dyspnea on exertion upon climbing ≤1 flight of stairs and abnormal breath sounds on auscultation); (8) pregnant patients or patients who are planning to become pregnant during the study; (9) recent (within 1 month) or anticipated treatment with corticosteroids (except those for short-term use during the time of chemotherapy) or other appetite stimulants; (10) bipolar disorder; (11) creatine supplementation within the past 30 days; and (12) a change in supplement use within the past 14 days.

### Allocation

Patients who are eligible to participate in the study will be allocated to either the creatine and exercise group or the exercise alone control group (1:1). Randomization will be performed through the study’s REDCap (Research Electronic Data Capture; Vanderbilt University) database. We will use randomized blocks to conceal the group allocation results from the patients, recruitment staff, and assessment staff, and concealment will be maintained until the patients complete the baseline assessments. The research pharmacy at the Mays Cancer Center will conceal the randomization and will direct the research team to assign study participants to specific groups. A sample of 15 breast cancer survivors will be enrolled in each group with the goal of having 12 completers in each group. Due to the nature of the study, the principal investigator and study participants will not be blinded. However, to alleviate any issues with assessment fidelity, the research assistant who will be performing the outcome assessments will not be aware of the groups to which participants are randomized. Further, the statistician who will be supporting the analyses in the study will be blinded as well.

### Exercise Intervention

All participants will engage in 3 center-based exercise sessions each week for 12 weeks. Each session will last roughly 1 hour and include a 10-minute warm-up and a 50-minute stimulus phase. Exercise sessions will be held on nonconsecutive days to allow for adequate rest and recovery. The prescription will include 2 or 3 sets of 6 exercises, which will be performed at a 10-repetition maximum intensity (ie, participants will be able to perform only 8 to 12 repetitions per set), as per the American College of Sports Medicine’s guidelines for exercise testing and prescription [29] and the consensus statement on exercise guidelines for cancer survivors [30]. Each session will consist of the following exercises, which will be done in the following order: chest press, leg press, seated row, shoulder press, leg extension, and leg curl. The resistance load will be set at 70% of the measured or estimated 1-repetition maximum. If participants cannot perform an exercise with this resistance load, the load will be reduced to allow them to complete the prescribed repetitions for each set. Participants will complete 2 sets of each exercise in week 1. This will allow participants to familiarize themselves with the resistance load and will minimize the risk of injury for novice participants. Starting in week 2, participants will transition to performing 3 sets of each exercise. The load will be increased when a participant is able to perform 12 repetitions in 2 of 3 sets for an exercise. Participants’ heart rates will be monitored continuously during exercise sessions. Self-reported perceived exertion will also be recorded periodically during exercise sessions [31], and this information will be used to track the subjective experiences of participants and interpret adherence data.

### Creatine Dosing Protocol

Participants who are randomized to receive creatine (experimental group) will be initially dosed at 20 g per day for 7 days to boost the availability of creatine systemically [32]. Thereafter, the dose will be reduced to 5 g per day for maintenance throughout the duration of the 12-week protocol. This dosing protocol is based on the traditional dosing protocol for healthy individuals, which includes a loading phase (5 g of creatine taken 4 times per day) that is followed by a low-dose maintenance period (5 g of creatine per day) for maintaining creatine stores [12]. The individuals in this group will receive creatine in powder form at 4 different schedules based on their progress throughout the protocol. Each bottle of creatine will contain a 5-mg dosing spoon. Bottle 1 will be provided at the start of the study, and an equivalent dose for the loading phase will be assigned in week 1 of the study. In week 2, participants will return to the clinic and be provided with 21 days’ worth of creatine doses (5 g/day). At weeks 5 and 9, participants will again return to the clinic and be given 28 days’ worth of creatine doses (5 g/day). Bottles will be exchanged at these scheduled intervals, and dosing adherence will be determined by measuring the amount of creatine that remains in the bottles. For weeks 2 to 12, participants will also be asked to take the creatine at approximately the same time of the day and enter the time of dosing in a dosing diary. The individuals in this group will have safety labs drawn after the loading phase to ensure normal kidney function, and electrocardiography will be performed to rule out any abnormalities. Participants will be removed from the creatine group if the lab results show serum creatinine levels of >2 times the upper limit of normal or estimated glomerular filtration rates of <30 mL/min/1.73 m^2^. These participants will complete the remaining exercise interventions.

### Study Procedures

#### Telephone Screening

Potential participants will undergo telephone screening to rule out clear, excluding medical conditions prior to in-person screening. Individuals who are known to meet no clear exclusionary criteria will be invited to an initial screening visit.

#### Screening Visit

At the screening visit, participants will read and sign the informed consent form. Once consent is provided, a complete medical history and physical exam will be performed to ensure that the participants meet the inclusion criteria for the study. This will include measurements of vital signs, height, and body weight. Blood will be collected following an overnight fast for clinical lab tests, which will include renal function testing, comprehensive metabolic panels, and lipid and complete blood count tests. Resting electrocardiograms will be acquired and evaluated by a clinician to confirm a healthy heart rate and rhythm. Questions regarding the intent to become pregnant will be asked. A mood disorder questionnaire will be completed by participants and evaluated by study staff to assess for bipolar mood disorder. Subjects who meet the study eligibility criteria will be scheduled for study assessments.

#### Outcome Assessments

Assessments will be performed at baseline and at the end of the study (Table 2).

**Table 2:** Study assessments and methods.

Assessment	Method
Renal function testing, metabolic panels, and lipid and complete blood count tests	Lab test
Muscle strength (power and torque)	Dynamometry
Muscle strength	1-repetition maximum and 10-repetition maximum training
Body composition and bone density	Dual x-ray absorptiometry
Muscle cross-sectional area	Magnetic resonance imaging
Muscle creatine, phosphocreatine, and adenosine triphosphate	Magnetic resonance spectroscopy
Physical function	6-minute walk test
Quality of life	European Organization for Research and Treatment of Cancer Quality of Life Questionnaire-C30 and European Organization for Research and Treatment of Cancer Quality of Life Questionnaire-BR23

#### Body Composition

Body composition (fat mass, muscle mass, and bone) will be measured via dual x-ray absorptiometry. By using dual x-ray absorptiometry scans, we will measure whole-body and regional adipose and lean mass [33] as well as bone mineral density.

#### Strength and Physical Function

A 1-repetition maximum test will be performed to evaluate the maximum amount of force that can be generated through a full range of motion for a particular body region [34]. This will be directly tested for chest presses and leg presses and estimated (via 10-repetition maximum testing) for shoulder presses, leg extensions, and leg curls. Isometric knee extensor strength will be assessed in the right leg of each participant (unless contraindicated) by using a Biodex dynamometer (Biodex Medical Systems). The isometric maximum voluntary contraction force will be measured at approximately 60° knee flexion [35]. The average values from 3 attempts and percent variance will be recorded. Dominant isometric handgrip strength will also be tested by using a handgrip dynamometer (Jamar Plus+ Digital Hand Dynamometer; Jamar Plus+) [36]. Participants’ 6-minute walk distances will be assessed to measure endurance function by using a standardized protocol [37].

#### Patient-Reported Outcomes

Study participants will complete the European Organization for Research and Treatment of Cancer Quality of Life Questionnaire (EORTC QLQ)-C30 (version 3) quality of life survey and EORTC QLQ-BR23 survey for breast cancer survivors [38,39]. The EORTC QLQ-C30 is a 30-item measure of health-related quality of life. It includes 6 functional scales (physical, role, emotional, social, cognitive, and global quality of life scales), 3 symptom scales (fatigue, pain, and nausea and vomiting scales), and 6 single items (dyspnea, sleep disturbance, appetite, diarrhea, constipation, and financial difficulties). The EORTC-QLQ-BR23 is a 23-item measure of the symptoms and problems experienced by breast cancer survivors.

#### Muscle Cross-sectional Area and In Vivo Assessments

Magnetic resonance imaging will be used to measure the right midthigh muscle cross-sectional area (unless contraindicated) by using a whole body 3.0 T magnetic resonance imaging scanner (TIM Trio; Siemens AG). At the same time, intramuscular creatine, phosphocreatine, and ATP content will be assessed in vivo by conducting magnetic resonance spectroscopy (MRS), as previously described [40,41]. A phosphorus-31/hydrogen-1 dual-tuned, rigid, arc-shaped surface coil (RAPID Biomedical GmbH) will be positioned under the vastus lateralis. The vastus lateralis muscle was chosen for in vivo analysis because of the superficial positioning of the muscle and because it is the largest muscle in the quadriceps muscle group, which is the largest and strongest muscle group in the body. Lower extremity muscle weakness is also associated with long-term fatigue in breast cancer survivors [3]; thus, in vivo measurements are warranted for this muscle. An external reference 6-mL plastic vial with an 850mM concentration of methylenediphosphonic acid will be fixed to the center of the coil. Methylenediphosphonic acid was chosen due to its resonance frequency of around 22 parts per million downfield from phosphocreatine; this does not overlap with any relevant metabolite peaks. After a subject is scanned, a 15-cm–diameter, 4-L, plastic, cylindrical leg phantom containing 35mM phosphoric acid will be placed on the coil and scanned by using the same MRS parameters and slab positions, so that the data can be collected from the same area within the radio frequency excitation field of the coil. A slice-selective phosphorus-31 MRS slab sequence (excitation pulse repetition time=10 seconds; time to echo=2.3 seconds; number of signals averaged=16; slice thickness=25 mm; receiver bandwidth=3000 Hz) will be performed for the quadriceps muscles of subjects and for the previously described leg phantom. Raw spectral data will be analyzed with Java Magnetic Resonance User Interface software [42], and the processing steps will include apodization to 5 Hz and Fourier transformation and phase correction. For spectrum quantification, the AMARES (Advanced Method for Accurate, Robust, and Efficient Spectral Fitting) algorithm [43] will be used.

### Capturing and Monitoring Adherence

Adherence to creatine supplementation will be assessed by measuring the quantity of creatine that is left in the returned bottles, as described in the *Creatine Dosing Protocol* section. Individuals will not be withdrawn from the study for noncompliance with the creatine dosing regimen. The dosing quantity (number of scoops) and the dosing time will be documented in a dosing diary. Each exercise session will be documented, and information regarding session duration time as well as exercise mode and intensity during each training session will be documented. Participants will be encouraged to adhere to the protocol to the best of their ability. Participants will be withdrawn if more than 33% (12/36) of exercise sessions are missed.

### Monitoring Adverse Events

Adverse events will be assessed during each interaction with a participant (ie, 3 times per week). Before and after each exercise session, participants will be asked about how they are feeling. If the answer is anything other than “feeling fine,” the participant will be queried about the date and time of the onset of an adverse event, self-perceived severity, and any self-administered treatments. Upon the conclusion of the intervention, each adverse event will be graded by medical professionals based on severity, the event’s relationship to the study intervention, the actions taken by the study personnel and participants, adverse event outcomes, whether the event was expected, and whether the event was serious. We will use the Common Terminology Criteria for Adverse Events version 5.0 [44] to grade the adverse events.

### Data Management

Each participant will be assigned a unique study code. Data will be saved and stored by using the REDCap electronic data management system. Surveys will be completed electronically and linked to the participants’ electronic data files to limit the incidence of transcription errors. Patients’ charts will be kept inside a locked cabinet that is located inside the clinical research space, which is directed by DIP.

### Statistical Analysis

The proposed analysis of 30 participants is based on a feasibility assessment [45]. Based on a preponderance of published feasibility and pilot studies, a minimum sample size of 15 participants per group was chosen to complete the study and estimate parameters for future studies [46,47].

Data on recruitment, adherence, and attrition rates will also be collected. Participants that withdraw from the study or are withdrawn for safety reasons will not be replaced.

Statistical analyses will be performed by using IBM SPSS 19.0 software (IBM Corporation). Descriptive statistics will be used to summarize the data. The primary analysis will be an intent-to-treat analysis, which will be conducted by using a 2-sided, stratified log-rank test. Cox proportional hazard regression models will be used to estimate hazard ratios with 95% CIs to quantify the effect of the intervention after adjusting for stratification, age, and BMI. We will also conduct secondary analyses for nonadherence, the rate of perceived exertion, and the number of exercise sessions completed. Pearson product moment correlation analyses will be used to measure associations among biomarkers of body composition, physical function, and quality of life measures. A *P* value of <.05 will be considered statistically significant, and all statistical tests will be 2-tailed. The results of our study will be used to calculate power and determine sample sizes for future research in this field.

## Results

As of October 2021, a total of 19 patients have been screened, 12 have consented, and 9 have enrolled into the study. No adverse events have been reported in the creatine arm of the study. Muscle soreness and tingling associated with chemotherapy-related peripheral neuropathy have been reported as adverse events. No unexpected adverse events have been reported. Enrollment is planned to be completed by summer 2022.

## Discussion

Our project will determine if creatine—a naturally occurring substance in the body—that is consumed as a supplement can be used to improve the adaptation of exercise training and accelerate the gain of muscle mass, strength, and lean body mass. Individuals with cancer are exposed to a variety of factors that impact their ability to live active lifestyles. Some of these factors are tumor related [48], while others are related to cancer treatment [49]. The loss of muscle mass and function greatly impacts a breast cancer survivor’s quality of life [50]. The prevalence of muscle loss in individuals with cancer can range from 11% to 74% [51-56]. Exercise has been shown to combat this decline in muscle mass [56]. Exercise programs are capable of attenuating many of the deleterious effects of muscle loss [56]. Commercially available agents, such as creatine, have the potential to accelerate exercise adaptations and increase muscle mass, strength, and lean body mass [57,58].

Only 4 studies have been published to date on the effects of creatine supplementation in cancer survivors [22-25], and only 1 of the 4 studies involves creatine dosing in combination with an exercise program. In the combination study, which was conducted by Lønbro et al [22] in 2013, a total of 30 patients with head and neck cancer who were treated with radiotherapy were supplemented with 5 g of creatine per day. The investigators reported greater improvements in lean body mass for creatine-supplemented participants compared to those reported for participants who took placebos [22]. A limitation to the study by Lønbro and colleages [22] was that they did not use the suggested dosing regimen (20 g of creatine per day) at the start of the study to load intramuscular creatine concentrations. Research has demonstrated that this loading protocol can result in increased intramuscular creatine stores (ie, a 10% to 40% increase in muscular creatine and phosphocreatine stores). Using a lower dose will result in a gradual increase. Our study will be the first to investigate the effects of creatine supplementation in combination with exercise by using the recommended dosing protocol.

There is strong evidence that suggests that creatine supplementation can promote the overexpression of genes and proteins related to muscle hypertrophy [59] and satellite cell activation [60] in healthy subjects who participate in an exercise program. The growth-promoting potential of creatine may be useful in situations where anabolic activity is suppressed, as is the case with patients with prostate cancer on androgen deprivation therapy [61]. Therefore, we hypothesize that a positive impact of our study will be a significant improvement in intramuscular stores of creatine that will result in greater improvements in muscle strength, physical performance, and lean body mass compared to those improvements in the control group.

## Abbreviations

AMARES: Advanced Method for Accurate, Robust, and Efficient Spectral Fitting
ATP: adenosine triphosphate
EORTC QLQ: European Organization for Research and Treatment of Cancer Quality of Life Questionnaire
MRS: magnetic resonance spectroscopy
REDCap: Research Electronic Data Capture
SPIRIT: Standard Protocol Items: Recommendations for Interventional Trials
